# Replacement of the ascending aorta and aortic valve for annuloaortic ectasia with Carbomedics Carbo-Seal valsalva™ graft: mid- to long-term results

**DOI:** 10.3389/fsurg.2025.1714007

**Published:** 2026-01-06

**Authors:** Raif Cavolli, Dogan Kahraman

**Affiliations:** 1 Cardiovascular Surgery Department, United Hospital, Prishtine, Kosova; 2 Department of Cardiovascular Surgery, SBU Kocaeli Derince Research and Training Hospital, Kocaeli, Türkiye

**Keywords:** annuloaortic ectasia, aortic root replacement, aortic valve, carbomedics carbo-seal valsalva TM graft, modified Bentall procedure

## Abstract

**Purpose:**

The modified Bentall procedure utilizing the Carbomedics Carbo-Seal Valsalva™ graft can be employed to address aortic root pathologies. In this study, we examined the performance of this conduit specifically for treating isolated annuloaortic ectasia. Our objective was to evaluate the long-term outcomes of these surgeries.

**Methods:**

A total of 48 consecutive patients with annuloaortic ectasia underwent aortic root replacement using the Carbomedics Carbo-seal Valsalva™ graft between 2012 and 2024. In 7 patients, additional cardiac procedures were performed: two underwent mitral valve annuloplasty, and five had coronary artery bypass grafting. The mean cardiopulmonary bypass time and aortic clamp time during the modified button-Bentall operations were 151 ± 37 min and 128 ± 14 min, respectively.

**Results:**

The operative mortality rate was 2.1% (*n* = 1). Late mortality was 6.3% (*n* = 3), with causes including chronic heart failure (2.1%; *n* = 1), cerebral hemorrhage (2.1%; *n* = 1), and pulmonary complications (2.1%; *n* = 1). Major late complications included cerebral hemorrhage (4.2%; *n* = 2), pulmonary bleeding (2.1%; *n* = 1), and gastrointestinal hemorrhage (2.1%; *n* = 1). The Kaplan–Meier estimated survival rates were 96.22% at 5 years and 95.20% at 10 years. Additionally, the Kaplan–Meier curves showed event-free survival rates of 98% at 5 years and 82% at 12 years (95% CI).

**Conclusions:**

Modified button-Bentall operations for annuloaortic ectasia, with Carbomedics Carbo-Seal Valsalva™ graft, can be performed with a low mid- and long-term mortality and morbidity.

## Introduction

Bentall and DeBono were the first to report the use of a composite graft and valve to replace an ascending aortic aneurysm and aortic valve over 50 years ago. For coronary artery reattachment, they employed circumferential suture lines around the coronary ostia and wrapped the native aortic wall around the prosthesis to control bleeding (1). However, evidence of an increased risk of pseudoaneurysm development, as well as coronary compression or detachment due to oozing within the perigraft space, led to the introduction of technical modifications, such as the Cabrol modification (2). It is important to note that the Cabrol modification carries a risk of thrombosis in the interposed Dacron conduits. Today, the most commonly performed technique for root replacement is the “button” technique developed by Kouchoukos and colleagues (3, 4). This approach has proven effective in avoiding tension on the coronary anastomoses, subsequently preventing excessive bleeding and kinking of the coronary arteries.

In this study, we aim to share our experiences accumulated over more than 10 years using the Carbomedics Carbo-Seal Valsalva™ graft. This graft is created by integrating a standard Carbomedics bileaflet mechanical valve with a Valsalva vascular graft, designed for simultaneous aortic valve replacement (AVR) and aortic root replacement, with or without the replacement of the ascending aorta. The woven Dacron graft is treated with cross-linked gelatin to ensure low porosity and effective sealing.

This surgical approach involved certain modifications to the traditional Bentall procedure, and we closely monitored the outcomes of our patients. Our objective was to better understand the effectiveness and safety of the Carbomedics Carbo-Seal Valsalva™ graft in this operation. We specifically focused on the survival rates of older patients and the occurrence of significant complications during this period.

We believe that this study will help bridge the gap in the limited long-term, conduit-specific data available for the Carbo-Seal Valsalva™ graft in cases of annuloaortic ectasia.

### Patients and methods

This retrospective observational study was approved by the ethics board and conducted following the Declaration of Helsinki. All patients were informed about the procedure and provided written consent for the operation.

The study was carried out from September 2012 to January 2024 at two different centers, with the same operative team and primary surgeon. A total of 48 patients underwent modified Bentall procedure (MBP; simultaneous aortic root and aortic valve replacement) using the Carbomedics Carbo-Seal Valsalva™ graft. This graft is utilized for patients requiring the simultaneous replacement of both the aortic valve and the ascending aorta, particularly those with annuloaortic ectasia. While none of the patients were definitively diagnosed with Marfan syndrome, five individuals (10.4%) exhibited characteristics consistent with Marfanoid features. Patients requiring non-elective surgery, such as those with type A aortic dissection or endocarditis, were excluded from the study. Preoperative aortic dimensions were measured using echocardiography and computed tomography.

The primary indication for surgery was an aneurysm of the ascending aorta associated with valve pathology. Additionally, a bicuspid aortic valve was identified in nine patients (18.8%).

Data were gathered from hospital records and patient files. Patient characteristics, including age, gender, comorbidities, and risk factors related to metabolic and cardiovascular diseases, were documented (Table 1).

**Table 1:** Preoperative characteristics of the patients.

Preoperative characteristics	*N* ^a^
Age	61.64 ± 5.85
Gender (M)	40 (84%)
NYHA functional class	
I	0
II	7 (14.6%)
III	29 (60.4%)
IV	12 (25%)
Aortic regurgitation (n)	
Moderate	20 (41.6%)
Advanced	28 (58.3%)
Stenosis + Regurgitation	6 (12.5%)
Bicuspid aortic valve	9 (18.8%)
Marfanoid patients	5 (10.4%)
Preoperative aortic dimensions(mm)	
Aortic annulus	27.5 ± 5.4
Aortic root	49 ± 6.6
Sinotubuler junction	54 ± 7.2
Ascending aorta(cm)	63,6 ± 8
EuroSCORE II	6.1 ± 2.3
Left Ventricular Ejection Fraction	
≥60%	16 (33.3%)
40–59%	27 (56.2%)
≤39%	5 (10.4%)
Coronary artery disease	5 (10.4)
Chronic obstructive pulmonary disease	1 (2.1%)
Atrial fibrillation	3 (6.2%)
Smoking	25 (52.1%)
Hypertension	26 (54.2%)
Cerebrovascular accident	3 (6.25%)
Diabetes mellitus	20 (41.6%)
Obesity (BMI>30)	12 (25%)
Chronic lung disease	15 (31.2%)
Myocardial infarction	5 (10.4%)
Chronic renal failure	2(4.2%)

a Data expressed as *n* (%) or mean ± SD.

NYHA, New York Heart Association; BMI, body mass index.

### Operative technique

All surgical procedures involved a median sternotomy, and each operation was conducted using cardiopulmonary bypass (CPB). CPB was established through atrial drainage and cannulation of the distal ascending aorta, in most cases, or the right axillary artery in cases where the proximal arch was affected, for arterial return. We preferred central cannulation over femoral cannulation to avoid complications such as retrograde embolism. We favored direct insertion of the cannula into the axillary artery rather than sewing a side-arm graft.

For 4 patients, circulatory arrest (CA) was implemented when the vesical temperature ranged from 22 to 26 °C. Moderate hypothermia was maintained with antegrade cerebral perfusion (ACP) to protect the brain during CA. In all cases, bilateral invasive blood pressure monitoring through the radial arteries was mandatory. Intraoperative cerebral monitoring was provided using near-infrared spectroscopy (NIRS).

For cases requiring arch repair, we performed the distal anastomosis openly while utilizing hypothermic CA and selective ACP. Myocardial protection was achieved through antegrade, retrograde, or a combination of both forms of intermittent cold hyperkalemic blood cardioplegia.

Composite conduits were implanted using a consistent technique throughout the study period. A valved conduit of appropriate size was implanted at the aortic annulus using 11 to 14 interrupted U-sutures with pledgeted 2-0 polyester, following the routine excision of the native valve leaflets and preparation of the coronary buttons. The model used in all patients was the Carbomedics Carbo-Seal Valsalva™. The remaining aortic skirt was secured to the sewing ring with an additional running 3-0 Prolene suture to enhance the hemostatic barrier.

**Figure 1:**
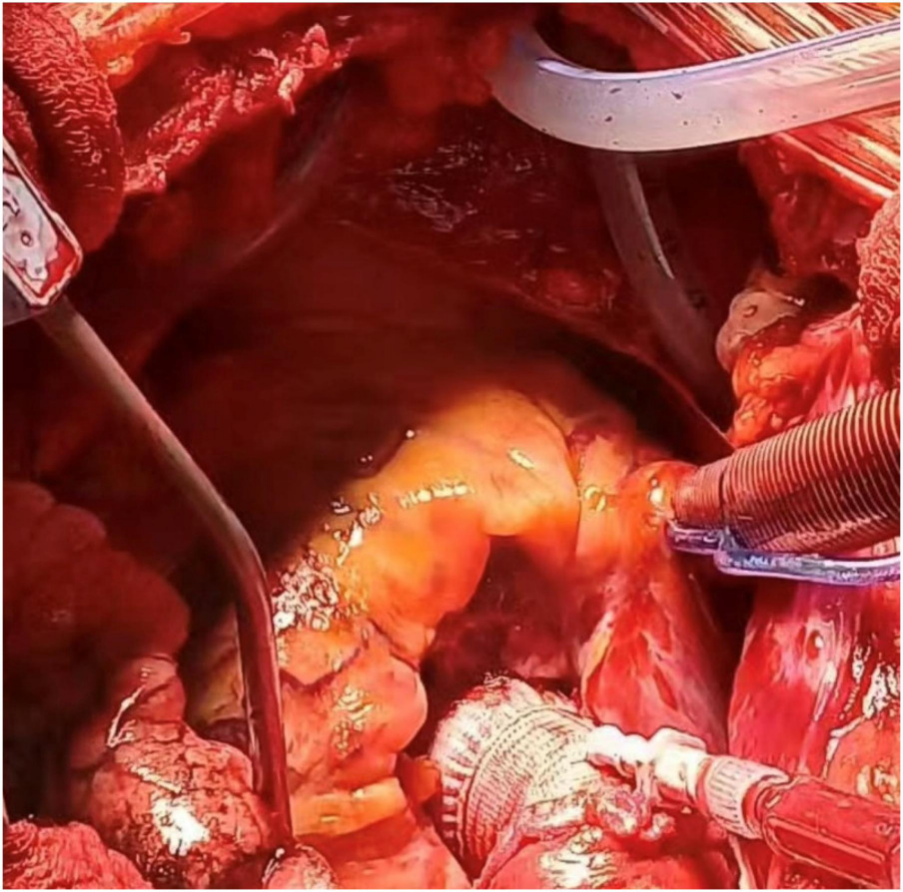
Intraoperative image of bentall procedure with carboSeal valsalva™ composite conduits.

First, the left coronary button was attached to the graft, followed by the right coronary button, using running 5-0 Prolene sutures in a standard fashion. The distal end of the graft, cut to the appropriate length, was sewn to the aorta with 4-0 Prolene and an externally positioned Teflon strip. Just before removing the aortic clamp, the cardioplegia cannula was inserted into the graft and connected to a vacuum line to create a bloodless field while fibrin glue was applied to all suture lines. Deairing maneuvers, rewarming, and weaning from CPB were performed as usual (Figure 1).

### Postoperative follow-up

Before leaving the hospital, all patients underwent a clinical evaluation, which included routine laboratory tests and a transthoracic echocardiogram. After discharge, patients were instructed to return for follow-up visits at various intervals: 1 month, 3 months, 6 months, and 1 year. The total follow-up time included 1,963 patients per year. Long-term anticoagulation was maintained with a target international normalized ratio (INR) of 2-2.5. Patients were specifically informed about the need to receive a computerized tomography (CT) angiography within the first year after the procedure.

### Study endpoints

The study endpoints include: (1) mid- and long-term mortality; (2) mid- and long-term morbidity; and (3) in-hospital freedom from all-cause major adverse events, as defined by established guidelines. Additionally, during long-term follow-up, it was recorded whether patients developed any documented non-cardiac co-morbidities that resulted in death.

### Statistical analysis

Continuous data are expressed as mean ± 1 SD or median; categorical variables are reported as a percentage; a commercial statistical software package (SPSS for Windows, version 25.0, SPSS Inc, Chicago, Ill) was used for data analysis. Analysis of the actuarial survival curve was performed by use of the Kaplan–Meier estimation for freedom from death and freedom from major complications.

## Results

### Preoperative data

A total of 48 patients underwent a MBP. The baseline characteristics of these patients are summarized in Table 1. There were 40 males (84%) with a mean age of 61.64 ± 5.85 years. Preoperatively, 7 patients were classified as New York Heart Association (NYHA) functional Class II, 29 were in Class III, and 12 patients were in Class IV.

### Operative and early results of first month

A 64-year-old patient with undiagnosed von Willebrand disease underwent reoperation for bleeding, and he died on the sixth day due to multi-organ failure following multiple blood transfusions (2.1%). Carbomedics Carbo-Seal Valsalva™ composite grafts were used in all patients (100%). The sizes of the grafts used were as follows: 23 mm (8.33%; *n* = 4), 25 mm (62.5%; *n* = 30), 27 mm (25%; *n* = 12), and 29 mm (4.16%; *n* = 2).

A standard MBP was performed in the majority of patients (*n* = 44; 91.6%), while 4 patients (8.4%) underwent additional hemi-arch replacement. Concomitant procedures were carried out in 7 patients (14.58%): mitral valve repair in 2 patients (4.16%) and coronary artery bypass grafting in 5 patients (10,4%).

Cannulation of the ascending aorta was utilized in most patients (*n* = 42; 87.5%), but axillary artery cannulation was necessary in 6 patients (12.5%). Axillary artery cannulation was performed only if the arch was to be repaired or if the aneurysm involved the entire ascending aorta. Selective ACP and deep hypothermic CA were used in only 4 patients, which represented 8.33% of the total cases. The mean CPB time was 151 ± 37 min, while the mean aortic cross-clamp time was 128 ± 14 min. The average volume of drainage was 614 mL, with a minimum of 450 mL and a maximum of 800 mL. All patients, except for one with von Willebrand disease, did not require reoperation due to excessive bleeding, nor did they experience endocarditis, or pericardial effusion. One patient who underwent concomitant mitral valve repair and MBP surgery experienced a cerebellar ischemic attack at the end of the second week. Relevant medical treatment resulted in a complete recovery of neurological symptoms within 5 days. 3 patients required prolonged mechanical ventilation. Other perioperative characteristics are detailed in Table 2.

**Table 2:** Perioperative characteristics.

Perioperative parameters	*N* ^a^
Cross-clamp time (min.)	128 ± 14
Perfusion time (min.)	151 ± 37
Total circulatory arrest (min.)	25 ± 5
Bleeding (mL)	614 (min. 450 mL, max. 800 mL)
Hospital Death (*n*)	1 (2.1%)
Cause of hospital death	
Von Willebrand disease (*n*)	1 (2.1%)
30-day mortality (*n*)	1 (2.1%)
ICU stay (days)	4.2 ± 2.5
Hospital stay (days)	10.0 ± 4.4
Concomitant procedures	
Mitral valve repair (*n*)	2 (4.2%)
Coronary artery bypass surgery (*n*)	5 (10.42%)
Prosthesis size (mm)	
23	4 (8,33%)
25	30 (62.5%)
27	12 (25%)
29	2 (4,16%)

a Data expressed as *n* (%), median (range) or mean ± SD.

ICU, intensive care unit.

### Mid-term (5-year) and long-term (10-year) results

#### Mortality

**Figure 2:**
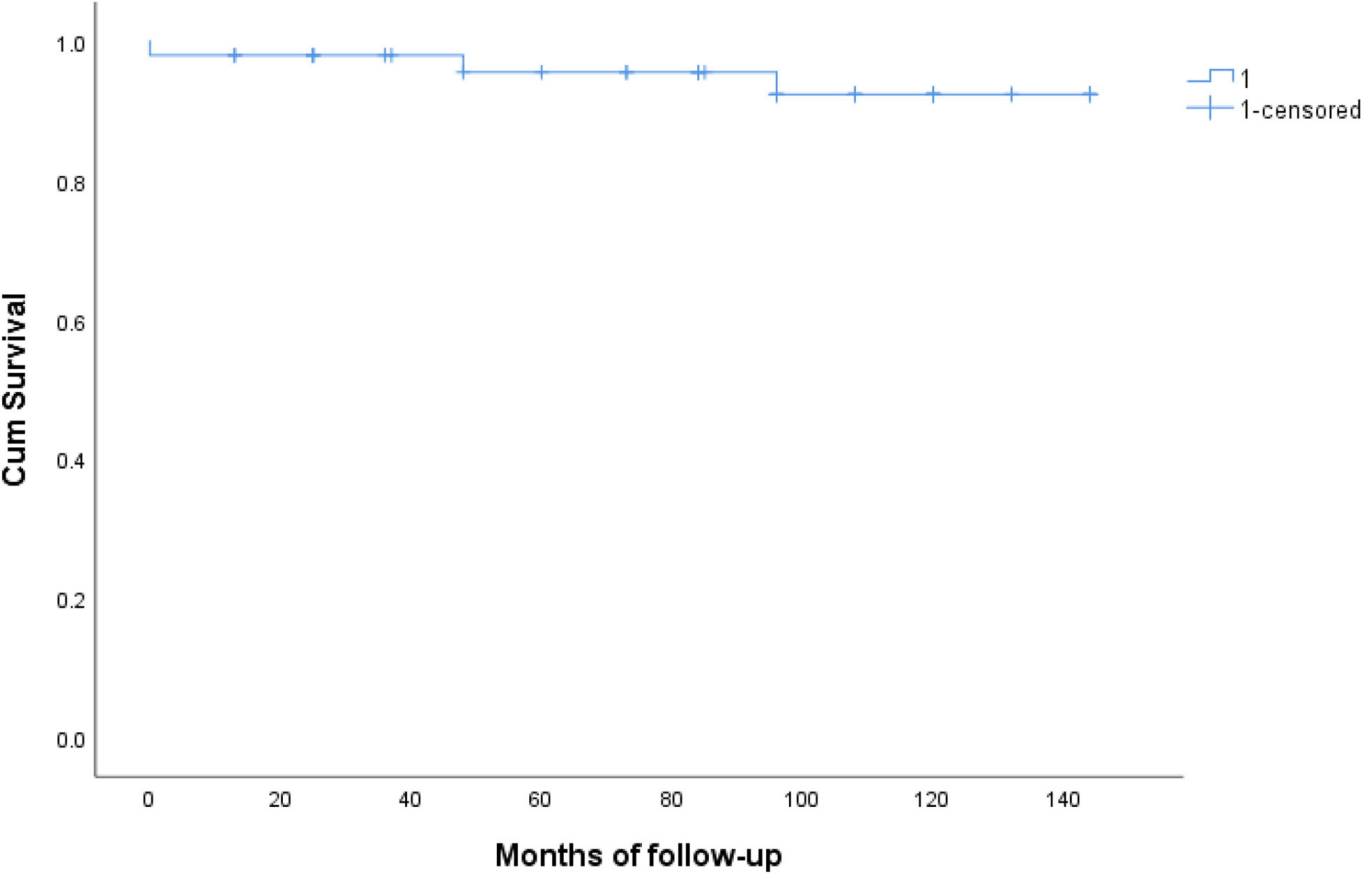
The results of the kaplan–meier analysis regarding freedom from all-causes death at 5 and 12 years were 97% and 93%.

**Figure 3:**
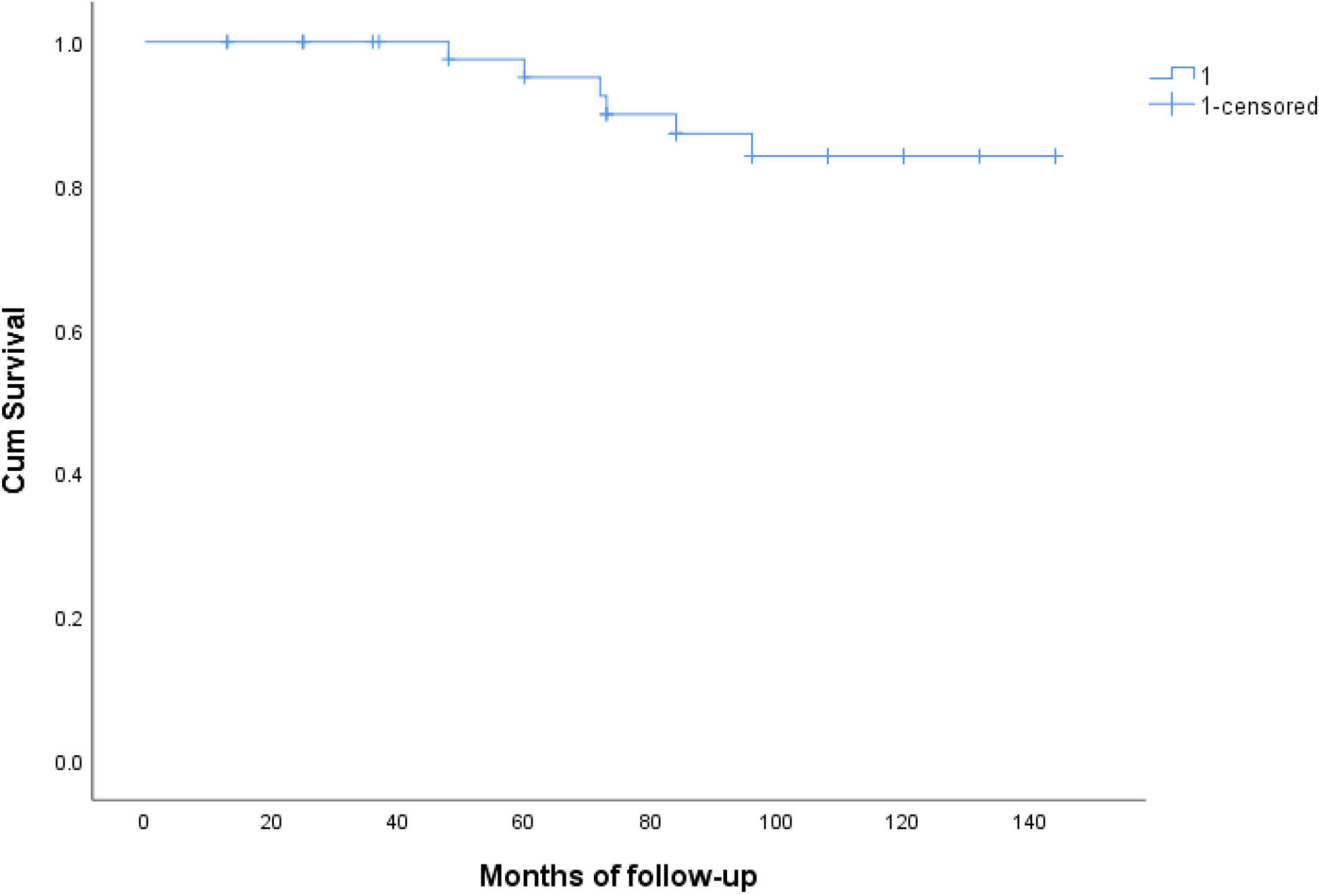
The kaplan–meier curves showed event-free survival at 5 and 12 years 98% and 82%, respectively (95% CI).

The summary of postoperative mortality is presented in Table 3. The overall mortality rate was 8.3% (*n* = 4). The causes of late mortality included chronic heart failure (*n* = 1), cerebral hemorrhage (*n* = 1), and chronic obstructive pulmonary disease (*n* = 1). There was no conclusive evidence of conduit dysfunction. Figure 2 illustrates the results of the Kaplan–Meier analysis, showing that the rates of freedom from all-cause death at 5 and 12 years were 97% and 93%, respectively (95% CI) (Figure 3).

**Table 3:** Early and late results.

30 day follow up	*N*
Mortality	1
Low cardiac output requiring IABP	3
Wound infection	
Superficial	2
Deep	0
Acute Kidney Injury (Crea. ≥1,5 × baseline)	4
New dialysis	0
Stroke	1
Reoperation	1
Prolonged ventilation ≥48 h	3
Late Results	1
Mortality	3
Chronic heart failure	1
Cerebral hemorrhage	1
Pulmonary complications	1
Valve dysfunction	0
Dissection/Rupture	0
Endocarditis	0
Warfarin-related events	
Cerebral hemorrhage	2
Pulmonary bleeding	1
Gastrointestinal hemorrhage	1

IABP, intra-aortic ballon pump; Crea, creatinin.

#### Morbidity

Postoperative complications are summarized in Table 3. During the follow-up period, the main challenge was establishing an appropriate INR target interval for patients who were not consistently adhering to scheduled visits.

Four patients required in-hospital treatment for bleeding, which included two instances of cerebral bleeding, one case of gastrointestinal bleeding, and one case of pulmonary bleeding. Throughout the long-term follow-up, patients were scheduled for echocardiographic examinations at a maximum one-year interval to assess the root functionality. There was no echocardiographic evidence of infective endocarditis or valve thrombosis.

Figure 2 presents the results of the Kaplan–Meier analysis related to freedom from all causes of late complications during the follow-up period. The Kaplan–Meier curves indicated an event-free survival rate of 98% at five years and 82% at twelve years, with a confidence interval of 95%.

## Discussion

Untreated aortic root dilation can lead to life-threatening complications, such as acute aortic dissection or rupture (5). To minimize the risk of these acute aortic events, current guidelines recommend elective replacement of the aorta and/or aortic root when the individually adjusted size threshold is reached (6). Moreover, performing the operation on the root earlier can enhance the chance of a successful valve-sparing procedure, especially in centers with significant experience (6, 7).

The Bentall procedure has long been regarded as the gold standard for surgically treating diseases of the combined aortic valve and ascending aorta. This technique, first described by Bentall and DeBono in 1968, involves the complete replacement of the ascending aorta and aortic valve along with the reimplantation of the coronary arteries (1). Over the years, the procedure has undergone numerous modifications as our understanding of various aortic root pathologies has advanced (3, 8). These modifications aim to achieve acceptable rates of morbidity and mortality; however, issues such as bleeding, perioperative myocardial infarction, and right ventricular dysfunction can still lead to death (8). Root replacement using a single composite graft offers several advantages, including the complete removal of the diseased aorta while preserving the functionality of the root, being a well-established surgical technique with reliable early postoperative outcomes, and worldwide access to different types of conduits (9).

The procedure carries inherent risks, including arrhythmia, heart failure, perioperative myocardial infarction, stroke, infection, excessive bleeding that may require reoperation, acute renal injury, pulmonary complications that necessitate prolonged ventilation, and the potential for new dissections or aneurysms (10). Though increasing expertise for decades has reduced these risks to an acceptable level, the incidence of pseudoaneurysms at the coronary artery reimplantation site has been reported to range from 3.1% to 9% (3, 11). We did not detect any coronary site pseudoaneursm in our patient cohort, and this result could be attributed to the neo-sinus design of the prosthesis allowing less dissection of button, less traction or kinking.

This retrospective study reports the mid- and long-term outcomes of 48 consecutive patients who underwent the Bentall procedure using the Carbomedics Carbo-seal Valsalva composite graft between 2012 and 2024. We believe that some unique structural and user-friendly features of this graft contributed to our favorable long-term results in this relatively small patient series. The vertical orientation of the Valsalva pleats allows for easier anastomosis of the coronary buttons and prevents longitudinal movement at the anastomosis point while adjusting the distal length of the graft. This innovative feature is designed to effectively mitigate issues such as traction, kinking, and rotation of the left coronary ostium. Furthermore, the elegantly crafted sinus design of the prosthetic device aims to encourage the formation of natural vortices, thereby enhancing blood flow and significantly lowering the risk of thrombosis (12, 13).

The overall mortality rate was 6.25%, and there was no need for reoperation due to cardiac issues such as infective endocarditis or valve dysfunction. Large series reporting the outcomes of Valsalva grafts generally have a heterogeneous patient population, including acute aortic syndromes and dissections to various extend (4). We believe that the differences in early and midterm results between our cohort and those found in the literature are primarily due to the diversity of pathology. The results we present from elective surgeries for chronic root dilation reflect the actuarial performance and durability of the Valsalva conduit. Our freedom from all-cause mortality at 12 years is 93%, and event-free survival at 12 years is 82%. These results are consistent with, and in some aspects comparable to, the best long-term outcomes of Bentall procedures reported in the literature (13).

Bleeding is a well-known and significant early complication following the surgery. The MBP involves several suture lines, each of which is at risk for postoperative bleeding, particularly in patients with fragile tissues, such as those with aortic dissection or Marfan syndrome. Therefore, techniques that ensure accurate hemostasis are essential for achieving a successful outcome and reducing postoperative morbidity. This is especially crucial at the venticuloaortic junction, where the composite conduit is secured. Once the aortic cross-clamp is released, any bleeding from the posterior aspect of the root becomes nearly impossible to repair (14). As a preventive measure, the application of fibrin glue has been used to seal this area. We believe that using vacuum suction within the graft while applying fibrin glue over the sutures is a more effective approach.

The MBP is likely to remain the standard treatment for this condition, even as valve-sparing procedures gain popularity for correcting aortic regurgitation due to aortic root pathology (15). The Bentall procedure may be the most suitable option, regardless of the underlying disease, especially in centers with a comparably smaller number of patients. This is because it requires less surgical expertise to achieve good results compared to valve-sparing operations, which involve a significant learning curve. Experiences indicate that the early onset of aortic insufficiency after a valve-sparing operation can lead to rapid and progressive hemodynamic deterioration, making it a riskier choice than the Bentall procedure, especially in patients with serious preoperative myocardial dysfunction. Additionally, patients with moderate or severe preoperative aortic insufficiency may have less favorable outcomes after valve-sparing surgery compared to those with mild aortic regurgitation (15–17).

In this study, all patients underwent a button Bentall operation, as described by Kouchoukos and colleagues. This procedure has the advantage of eliminating the need for coronary graft interposition, which can sometimes lead to complications such as kinking, coronary thrombosis, and early occlusion. Some complications arose later, and these were associated with anticoagulant therapy. Despite most patients being monitored by a cardiologist, we encountered six events directly related to anticoagulants. The introduction of home testing for coagulation status presents a new opportunity for better follow-up of patients undergoing mechanical valve replacement. Additionally, Schmidtke and colleagues demonstrated that self-management of anticoagulation results in a superior quality of life after mechanical valve replacement, compared to conventional physician-monitored anticoagulation (18).

### Limitations

When analyzing the findings of this study, it is important to acknowledge some limitations. Firstly, this study was conducted retrospectively. Additionally, it focused on only one team and did not include a control group. Consequently, the study was limited by the relatively small number of patients involved. Despite these limitations, our research provides valuable long-term outcomes for patients who underwent aortic root replacement using Carbomedics Carbo-Seal Valsalva™ composite grafts.

## Conclusion

In conclusion, the study demonstrates that the rates of mortality and morbidity following MBP with Carbomedics Carbo-Seal Valsalva™ composite grafts over an extended period are comparable to those reported in other research studies. The results indicate that MBP can achieve positive outcomes both in the short term and the long term. However, complications related to anticoagulation remain a significant challenge that must be addressed effectively. It is essential to carefully prevent and manage these complications to ensure patient safety.

## Data Availability

The raw data supporting the conclusions of this article will be made available by the authors, without undue reservation.
